# Ovarian response after random-start controlled ovarian stimulation to cryopreserve oocytes in cancer patients

**DOI:** 10.5935/1518-0557.20180065

**Published:** 2018

**Authors:** Ana Paula CB Campos, Guilherme P Geber, Rodrigo Hurtado, Marcos Sampaio, Selmo Geber

**Affiliations:** 1 UFMG – Universidade Federal de Minas Gerais, Belo Horizonte/MG, Brazil; 2 ORIGEN – Center for Reproductive Medicine, Belo Horizonte/MG, Brazil

**Keywords:** random-start, controlled ovarian stimulation, oocyte vitrification, fertility preservation

## Abstract

**Objective:**

To evaluate COS and oocyte retrieval results in ART treatment cycles initiated at any stage of the menstrual cycle (random start) in cancer patients, who could not postpone the onset of cancer treatment.

**Methods:**

Prospective observational study of 26 women with cancer, with an indication to start cancer treatment within the next 20 days and wishing to preserve their fertility. Ovarian stimulation started immediately with FSH followed by GnRH antagonist for pituitary suppression and GnRH agonist for oocyte maturation. Treatment started from day 1 to day 14 of the menstrual cycle was considered to be in the follicular phase, and that started from day 15 to day 28 was considered to be in the luteal phase. Oocyte retrieval was performed 34 h after GnRH agonist administration. After identification and maturity classification, metaphase II oocytes were cryopreserved using vitrification.

**Results:**

A total of 13 women had breast cancer, 4 ovarian cancer, 3 Central Nervous System cancer, 3 endometrial cancer, 2 cervical cancer and one bowel cancer. Thirteen patients started treatment during follicular phase and 13 during luteal phase. We found similar results for the duration of treatment, total dose of follicle stimulating hormone, number of ampoules of gonadotropin releasing hormone antagonist, mean number of follicles identified at ultrasound on the day of trigger and retrieval, number of aspirated oocytes and Metaphase II oocytes.

**Conclusion:**

Random-start controlled ovarian stimulation for emergency fertility preservation for minimizing delay in oncologic treatment for cancer patients does not interfere with the number of metaphase II oocytes, and therefore can be routinely used for stimulation followed by cryopreservation.

## INTRODUCTION

Life expectancy after cancer treatment is increasing continuously, due to improved prognosis and higher survival rates. Some treatments such as surgery (oophorectomy), radio and chemotherapy can adversely affect female reproductive and endocrine functions, and one third of cancer patients will develop premature ovarian failure (Chung *et al*., 2013). Cancer patients at reproductive age might express the desire to have biological children and need fertility preservation before starting treatment.

The use of oocyte or embryo cryopreservation is the preferred method for fertility preservation, with clinically acceptable results (ASRM Practice Committee, 2013). Both alternatives require controlled ovarian stimulation (COS) to increase the number of embryos/oocytes for cryopreservation. COS is performed using gonadotropins associated to either gonadotropin releasing hormone (GnRH) agonists or antagonists, to suppress pituitary function. For women submitted to Assisted Reproductive Technology (ART) treatment, COS starts at early follicular or mid luteal phases, in order to optimize pregnancy rates. This treatment might require 14 to 30 days, depending on the patient’s menstrual cycle phase at the presentation day.

In many cases, there is an urgent need to start cancer treatment, and patients cannot delay the onset of treatment, and it is not possible to wait for the ideal time to start COS. Therefore, COS can be initiated at any stage of the menstrual cycle (emergency fertility preservation) (Cakmak *et al*., 2013). This alternative is possible as there are multiple waves of follicular recruitment throughout the menstrual cycle. Also, the use of GnRH antagonists associated to gonadotropins enables the induction of multifollicular development with no follicular rupture. Therefore, COS and oocyte retrieval for embryo/oocyte cryopreservation can be performed without compromising the cancer treatment. However, experience with the emergency fertility preservation protocol is still scarce and with conflicting results.

Thus, we performed this study in order to evaluate COS and oocyte retrieval outcomes in ART treatment cycles initiated at any stage of the menstrual cycle (random start) in cancer patients, who could not postpone the onset of cancer treatment.

## MATERIAL AND METHODS

We performed a prospective observational study between April 2013 and June 2016, in a private IVF clinic in Brazil. The study was approved by the research ethics committee of the Universidade Federal de Minas Gerais and by the Brazilian research ethics committee (Conselho Nacional de Ética em Pesquisa - CONEP - 16871). All women read and signed a written informed consent form. A total of 26 women with cancer and an indication to start cancer treatment within the next 20 days, who wished to preserve their fertility, were included in the study. The age of the patients ranged from 21 to 40 years (mean 31.4±0.7) and all patients had normal menstrual cycles within the last 6 months.

A total of 13 women had breast cancer, 4 had ovarian cancer, 3 had Central Nervous System cancer, 3 had endometrial cancer, 2 had cervical cancer and one had bowel cancer. Twenty-three patients were nulligravidae and 3 had had previous miscarriages. The patients were separated into two groups according to the menstrual phase in which they started ovulation induction, i.e., Follicular Phase group (day 1 to day 14 of the menstrual cycle - n=13) and Luteal Phase group (day 15 to day 28 of the menstrual cycle - n=13).

### Ovulation induction and oocyte retrieval

Ovarian stimulation started immediately with recombinant follicle stimulating hormone (FSH) (Gonal-F; Merck Serono - Brazil) with doses ranging from 150 to 375 IU/day, according to the patient's age, in a step-down protocol. The dose was tailored according to the ovarian response measured by estradiol serum concentrations and follicular growth, as monitored by vaginal ultrasound. When a leading follicle achieved 14 mm, GnRH antagonist (cetrorelix; Cetrotide, Merck Serono - Brazil) was used for pituitary suppression. Oocyte maturation was induced with GnRH agonist (0.2 mg triptorelin - Gonapeptyl Daily; Ferring - Brazil) when >2 follicles reached a mean diameter of 17 mm.

When treatment started from day 1 to day 14 of the menstrual cycle it was considered to be in the follicular phase and when it started from day 15 to day 28 of the menstrual cycle it was considered to be in the luteal phase, according to the last menstrual period. Cycle phase was confirmed using vaginal ultrasound and measurement of serum estradiol, luteinizing hormone (LH) and progesterone levels.

Oocyte retrieval was performed ~34 h after GnRH agonist administration, by vaginal ultrasound guided aspiration. When necessary, follicles were flushed with HEPES buffered culture media (Sigma - USA). After identification, the oocytes were transferred to 20µL droplets of culture medium (Global Total - USA) covered with mineral oil (Sigma - USA) and incubated at 37^o^C and 6% CO_2_ for two hours. Metaphase II oocytes were confirmed by the presence of two pronuclei and were cryopreserved using vitrification.

### Vitrification

Vitrification was performed using the Cryotop method as previously described (Kuwayama *et al*., 2005). Briefly, the oocytes were balanced in a 7.5% (v/v) ethylene glycol + 7.5% dimethylsulfoxide (DMSO) solution in tissue culture medium (TCM199) +20% synthetic serum substitute (SSS), referred to as equilibrium solution (ES - INGAMED - Brazil) at 25ºC for 15 min. They were then placed into a vitrification solution that was the same as the ES, except that the concentrations were 15% ethylene glycol +15% DMSO + 0.5 M sucrose (INGAMED, Brazil). After 1 min in this solution, the embryos were placed on the Cryotop strip and immediately submerged into liquid nitrogen.

### Statistical analysis

Statistical analysis was performed using the *Mann-Whitney* test. The results are presented as mean±SD. Difference was considered significant when *p*<0.05.

## RESULTS

The mean age of the patients was similar in both groups. All patients received GnRH agonist to trigger oocyte maturation. When we compared treatment outcome from both groups, we found similar results for the duration of treatment, total dose of recombinant FSH, number of GnRH antagonist ampoules, mean number of follicles identified upon ultrasound on the day of trigger and retrieval, number of aspirated oocytes and Metaphase II (Table 1).

**Table 1:** Results from patients submitted to controlled ovarian stimulation for emergency oocyte cryopreservation, starting in the Follicular or Luteal phase

	Follicular Phase n=13	Luteal Phase n=13	p*
Age (years)	32.8±1.4	30.3±2.6	0.460
Days of ovarian stimulation	10.6±2.1	10.0±0.4	0.481
FSH total dose	2,610±160	2,587±152	0.926
GnRH antagonist ampoules	4.6±1.2	4.7±0.3	0.508
Follicles at ultrasound	26.6±4.4	21.5±3.4	0.360
Aspirated Follicles	26.6±3.9	19.2±4.0	0.272
Oocytes	20.4±5.7	18.5±4.5	0.927
Metaphase II	10.0±3.4	13.7±3.2	0.410

* Mann-Whitney test.

## DISCUSSION

Our study demonstrated that random-start controlled ovarian stimulation for emergency fertility preservation is a valid strategy for minimizing delay in oncologic treatment for cancer patients. To our knowledge only a limited number of studies compared COS started during follicular and luteal phase for fertility preservation in cancer patients, with conflicting results. A recent meta-analysis included only 4 studies comparing treatment for fertility preservation with a total number of 115 patients (Boots *et al.,* 2016). Although we report a limited number of cases, our results might contribute to increase the experience with random-start COS for fertility preservation in cancer patients.

The total dose of gonadotropin used for COS was similar in both groups, which is in agreement with previous studies analyzing random-start COS. Von Wolff *et al*. (2009) performed a multicenter study with 40 cancer patients and a similar total amount of FSH used for patients starting COS on the follicular or luteal phase. Maman *et al*. (2011) evaluated 18 patients and also found similar need for gonadotropin in both phases. Cakmak *et al*. (2013) evaluated 35 cancer patients and reported that the total dose of gonadotropin used was significantly higher in patients starting COS during the luteal phase. Kim *et al*. (2015) evaluated 22 patients and also described higher doses of gonadotropin in patients starting during luteal phase. However, they only included 5 patients starting during the luteal phase. Boots *et al*. (2016) performed a meta-analysis and also described a higher dose of gonadotropins in patients starting during the luteal phase.

When we compared the COS duration between the two groups, we found similar values in both groups. The results were similar to those described by von Wolff *et al.* (2009) and Kim *et al*. (2015). However, Cakmak *et al*. (2013) and Boots *et al*. (2016) described higher lengths of time in the group that started during the luteal phase. This difference can be explained by the variation in the number of patients among the studies.

The number of retrieved oocytes and metaphase II oocytes did not differ between the patients who started COS during follicular the phase or the luteal phase. These results are in accordance with the ones described by other researchers (von Wolff *et al*., 2009; Maman *et al*., 2011; Cakmak *et al*., 2013; Kim *et al*. (2015)), who also described similar numbers of retrieved oocytes and metaphase II oocytes in the luteal phase group and in the follicular phase group. Boots *et al*. (2016) described similar numbers of total oocytes retrieved; however, they described a reduced number of metaphase II oocytes in the group of patients who started COS during the luteal phase.

In conclusion, our results demonstrate that random-start controlled ovarian stimulation for emergency fertility preservation, for minimizing delay in oncologic treatment for cancer patients does not interfere with the number of metaphase II oocytes and, therefore, can be routinely used for stimulation followed by cryopreservation. Moreover, it might have a significant impact on the management of women with cancer, providing more patients with the opportunity to undergo fertility preservation. Additional studies are necessary to confirm the efficiency of this new strategy regarding pregnancy and live birth rates.
